# Impact of thrombocytes, on bacterial growth and antimicrobial activity of selected antibiotics

**DOI:** 10.1007/s10096-019-03762-1

**Published:** 2019-12-01

**Authors:** Alina Karoline Nussbaumer-Pröll, Sabine Eberl, Birgit Reiter, Thomas Stimpfl, Walter Jäger, Stefan Poschner, Markus Zeitlinger

**Affiliations:** 1 Department of Clinical Pharmacology, Medical University of Vienna, Waehringer Guertel 18-20, 1090 Vienna, Austria; 2 Clinical Department of Medical and Chemical Laboratory Diagnostics, Medical University of Vienna, Vienna, Austria; 3 Divison of Clinical Pharmacy and Diagnostics, University of Vienna, Vienna, Austria

**Keywords:** Thrombocytes, MHB, TKC, MIC

## Abstract

In vitro pharmacodynamic models are used to optimize in vivo dosing regimens in antimicrobial drug development. One limiting factor of such models is the lack of host factors such as corpuscular blood components as erythrocytes which have already been shown to impact activity of antibiotics and/or growth of the pathogen. However, the impact of thrombocytes has not previously been investigated. We set out to investigate if the addition of thrombocytes (set to physiological concentrations in blood of healthy human, i.e., 5 × 10^5^ thrombocytes/μL standard growth media Mueller Hinton Broth, MHB) has an influence on bacterial growth and on the efficacy of antibiotics against Gram+ and Gram− bacteria. Growth assays and time-killing-curves (TKC) were performed with ATCC-strains of *Escherichia coli*, *Staphylococcus aureus*, and *Pseudomonas aeruginosa* in triplicate over 24 h. The same approach was followed for 5 clinical isolates of *Escherichia coli*. Meropenem, ciprofloxacin, and tigecycline were tested as representatives of broad-spectrum antibiotics, and concentrations several-fold above and below the minimal inhibitory concentration (MIC) were simulated. No significant impact of thrombocytes was found on bacterial growth or antimicrobial stability for the investigated agents. Bacteria reduced thrombocyte content to different degree, indicating direct interaction of pathogens and thrombocytes. Impact on bacterial killing was observed but was not fully reproducible when thrombocytes from different donors where used. While interaction of bacteria and thrombocytes was evident in the present study, interaction between antibiotic activity and thrombocytes seems unlikely. Whether variability was caused by different thrombocyte concentrates needs further investigation.

## Introduction

There are various in vitro models in existence; these include both static (MIC, MBC, and MPC) and dynamic (time killing) versions. What these models have in common is that they all attempt to link in vitro activity to in vivo efficacy in patients.

For one thing, it is often unclear which factors should be incorporated in these models to produce reliable results. Otherwise, there is the need to keep models technically feasible.

Therefore, the impact of potential factors on the interplay between pathogens and antimicrobial agents should be individually assessed. Host factors such as pH, protein binding, and cations, as well as corpuscular blood components such as erythrocytes have been shown to impact activity of antibiotics and/or growth of the pathogen [1–4]. Nevertheless, the impact of thrombocytes, which may be equally relevant, has not previously been investigated. Therefore, we set out to examine if the addition of human thrombocytes to standard growth media has an impact on bacterial growth of representatives of Gram-positive and Gram-negative bacteria, and on the antibiotic activity of broad-spectrum antibiotics with different chemical properties and biological behavior.

## Methods

The experiments including bacterial growth assays and TKC were performed with *S. aureus* (ATCC® 29213TM), *E. coli* (ATCC® 25922TM), and *P. aeruginosa* (ATCC® 27853TM) obtained from the American Type Culture Collection (ATCC) (American Type Culture Collection, Manassas, VA, USA) as well as with 5 clinical isolates of *E. coli*. As growth media, non-cation adjusted Mueller Hinton Broth (MHB) (Merck, Darmstadt, Germany) was used for all bacterial strains, containing 17.5 g/L casein acid hydrolysate, 2 g/L beef extract and 1.5 g/L starch. MHB was adjusted to a final pH of 7.4 ± 0.2 and calcium was added (sterile filtered) to a final concentration of 20 mM Ca2+ (MHB_Ref_). The adapted setting with thrombocytes was generated with filtered thrombocyte concentrates (final pH 7.1–7.2) for transfusion with a cell count of 1500 × 10^3^/μL which were obtained from the department of blood serology and transfusion medicine at the general hospital of Vienna (General Hospital, Vienna, Austria). The final concentration of the thrombocytes in our experiments was set to the physiological concentrations seen in the blood of a healthy human, i.e., 2.5 × 10^5^ thrombocytes/μL in MHB_Ref_. Adjusted MHB_Ref_ with thrombocytes will hereafter be referred to as MHB_Throm_.

Moreover, susceptibility testing and pharmacodynamic experiments were conducted with meropenem (trihydrate powder, Sigma-Aldrich, Steinheim, Germany), ciprofloxacin (parenteral infusion solution, Ciprofloxacin Kabi®, Fresenius Kabi Austria GmbH, Graz, Austria), and tigecycline (hydrate, Tygacil, Sigma-Aldrich, Steinheim, Germany).

In detail, the determination of the minimal inhibitory concentration (MIC) for all bacterial strains was completed according to the performance standards for antimicrobial susceptibility testing of the Clinical and Laboratory Standards Institute (CLSI) (National Committee for Clinical Laboratory Standards). All TKC and growth analyses were performed in 3 mL MHB_Throm_ or MHB_Ref_ in 14-mL falcon tubes in triplicates or duplicates over 24 h in a shaking water bath at 37 °C under aerobic conditions. Addition of thrombocytes was done prior to inoculation with the bacteria. The bacterial suspension was adjusted to 1.5 × 10^8^ cells/mL in NaCl, corresponding to a McFarland standard of 0.5, and was added to the test tubes at a final concentration of 1.5 × 10^6^.

Concentrations were simulated several-fold above and below the MIC with meropenem, ciprofloxacin, and tigecycline for *E. coli* (MIC 0.03; 0.015 and 0.125 mg/L*)*, *S. aureus* (MIC 0.06; 0.5 and 0.06 mg/L), and *P. aeruginosa* (MIC 0.5; 0.25 and 4 mg/L), respectively. The same approach was followed for the clinical isolates of *E. coli*.

Samples were taken at time point 0 h (before the addition of antibiotics), 3 h, 7 h, and 24 h. Subsequently, seven serial dilution steps were carried out in 96-well microtiter plates filled with 0.9% NaCl. Samples were dropped onto Columbia blood agar plates followed by 24 h incubation at 37 °C under aerobic conditions. Further, CFUs were counted and the CFU per milliliter was calculated by taking the dilution steps into consideration. This was done using the following equation: number of CFU multiplied by 5 × 10^*n*^, with *n* representing the dilution number.

To check the integrity of thrombocytes within TKC experiments, control samples of MHB_Throm_ and samples with additional bacterial suspension of *E. coli*, *S. aureus*, and *P. aeruginosa* at a final concentration of 1.5 × 10^6^ bacterial cells were generated in triplicates and incubated at 37 °C for 24 h. A quantitative analysis of intact thrombocytes was done with a hematology analyzer XE-5000 (Sysmex, Austria GmbH), before and after incubation. In addition, to evaluate stability of an antibiotic in the presence of thrombocytes, HPLC analysis was done to determine the quantity of free antibiotic in the different media. Analysis was done for MHB_Throm_ and MHB_Ref_, for ciprofloxacin and meropenem at a final concentration of 0.1 μg/mL and at 1 μg/mL for tigecycline. The procedure was performed in analogy to erythrocyte experiments recently published [4].

## Results

**Fig. 1:**
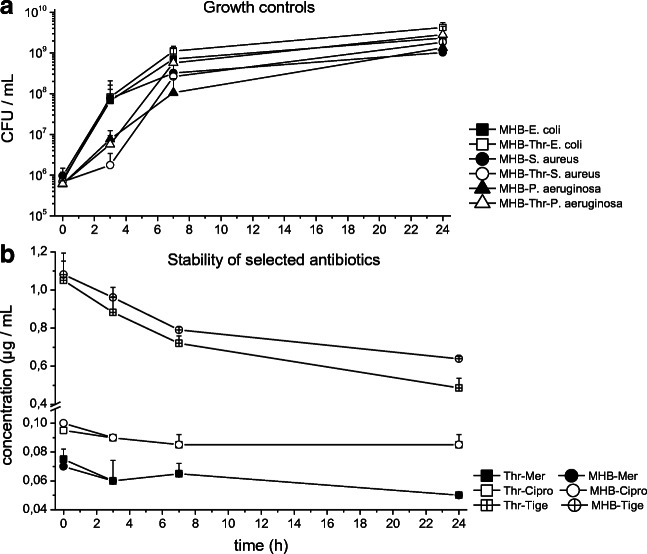
**a** The bacterial growth is represented in CFU per milliliter with MHB_Throm_ (open symbols) and MHB_Ref_ (black symbols) for *E. coli*, *S. aureus*, and *P. aeruginosa* over 24 h. **b** Concentration-time profiles of meropenem (black symbols), ciprofloxacin (white symbols), and tigecycline (crossed symbols) are shown in MHB_Throm_ (squares) and MHB_Ref_ (circles)

Our results show that for the growth of *E. coli*, thrombocytes had no significant impact. In the presence of thrombocytes, *P. aeruginosa* showed a slightly better growth at the 7-h time point, while *S. aureus* showed slightly impaired growth after 3 h compared with MHB_Ref_. Overall, no significant impact was present after 24 h (Fig. 1a).

Contrary to TKC experiments with erythrocytes, the impact of thrombocytes on bacterial killing varied strongly between the different settings but showed no reproducible image when the same setting was repeated several times. Nevertheless, within each experiment, higher concentrations always produced better killing than low concentration. This, together with the small standard deviations of the replicates, demonstrates the validity of the results within the study days.

**Fig. 2:**
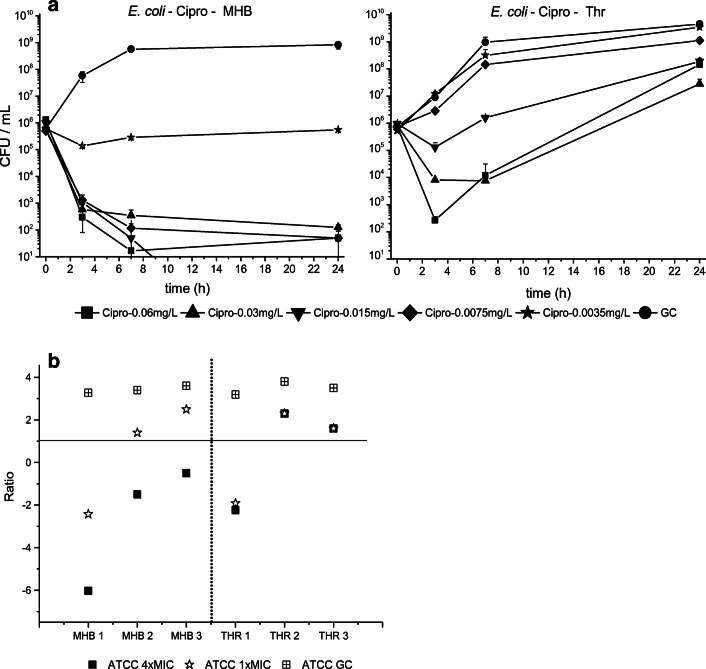
**a** The mean data with standard deviation of the TKCs of *E. coli* tested for ciprofloxacin are shown. **b** Variability of impact of thrombocytes on killing by ciprofloxacin presented as ratios of bacterial killing after 24 h between MHB_Ref_ and MHB_Throm_. Ratios above 1 indicate a decrease in antibiotic activity by addition of thrombocytes while values below 1 show increased antibiotic activity

Indeed, from TKC data shown in Fig. 2a, one might assume a significantly impaired activity of ciprofloxacin against *E. coli* in the presence of thrombocytes. Similar phenomena occurred as well for other combinations of the 3 tested strains and 3 antimicrobial agents (data not shown) but could not be sufficiently reproduced when experiments were repeated on different days.

To describe this variability, the impact of addition of thrombocytes on ciprofloxacin activity is presented for *E. coli* ATCC® 25922TM for 3 different study days in Fig. 2b. While on none of the days thrombocytes impaired bacterial growth, ratios of MHB_Throm_/MHB_Ref_ of log10 change CFU per milliliter compared with baseline indicate an increase in antibiotic activity in the presence of thrombocytes on 1 day, whereas on the two others, a decrease was found.

To evaluate the quality and stability of thrombocytes, counts of platelets were done at different time points. At time point 0 h, counts were comparable for all samples, with and without bacteria. In MHB_Throm_, the integrity of thrombocytes was only slightly (not significant) affected by incubation over 24 h at 37 °C with values of 3.49 × 10^5^/μL (SD ± 2.23 × 10^4^) at time 0 h and 2.73 × 10^5^/μL (SD ± 8.02 × 10^3^) at 24 h. Contrary, varying impact has been shown after incubation with bacterial strains (Table 1). The highest count of thrombocytes after 24 h was found with *P. aeruginosa* with 1.63 × 10^5^/μL (SD ± 3.61 × 10^3^), followed by *S. aureus* with 9.7 × 10^4^/μL (SD ± 5.69 × 10^3^) while for *E. coli* after 24 h only 8 × 10^3^/μL (SD ± 2.00 × 10^3^) thrombocytes remained.

**Table 1:** Average values of platelet counts over time with standard deviation from 0 to 24 h for MHB_Throm_ and for MHB_Throm_ adapted with *E. coli*, *S. aureus*, and *P. aeruginosa*

Average values of platelet count in THR per microliter				
Time point	MHB_Throm_	MHB_Throm_ *E. coli*	MHB_Throm_ *S. aureus*	MHB_Throm_ *P. aeruginosa*
0 h	3.49E+05 (± 2.23 × 10^4^)	3.05E+05 (± 2.65 × 10^3^)	2.99E+05 (± 2.65 × 10^3^)	3.17E+05 (± 2.27 × 10^4^)
3 h	3.26E+05 (± 1.33 × 10^4^)	8.00E+03 (± 2.52 × 10^3^)	1.83E+05 (± 3.06 × 10^3^)	1.91E+05 (± 2.65 × 10^3^)
7 h	3.01E+05 (± 2.65 × 10^3^)	8.00E+03 (± 2.08 × 10^3^)	1.01E+05 (± 3.61 × 10^3^)	1.82E+05 (± 1.53 × 10^3^)
24 h	2.73E+05 (± 8.02 × 10^3^)	8.00E+03 (± 2.00 × 10^3^)	9.70E+04 (± 5.69 × 10^3^)	1.63E+05 (± 3.61 × 10^3^)

Stability of meropenem and ciprofloxacin was not impacted by the presence of thrombocytes as shown in Fig. 1b. Tigecycline stability, on the other hand, showed slightly reduced stability in MHB_Throm_ compared with MHB_Ref_, and overall showed a stronger degradation over time than the other two antibiotics.

## Discussion

Since all TKC were conducted precisely under the same laboratory conditions, the only source of variability was the thrombocyte concentrates. We must keep in mind that the thrombocytes used were not from one homogeneous thrombocyte concentrate but different thrombocyte concentrates from different donors were used. For the sake of their integrity these concentrates could not be frozen and pooled to a later time point but had to be processed within a certain time period. Inter-individual differences and quality of the concentrates might therefore be an important source of our observed variation.

Compared with erythrocytes, which account for approx 40–50% of volume in blood, thrombocytes account for what is essentially a negligible volume percentage, both at physiological concentrations in blood and in our experiments. Therefore, for thrombocytes, the observed impact on bacterial killing might rely on pharmacodynamic interaction rather than pure intracellular binding. Enzymatic degradation might play a role in settings with reduced activity, since degradation of tigecycline was promoted in the presence of thrombocytes (Fig. 1b). Importantly, in contrast to erythrocytes, thrombocytes might undergo conformational change in the presence of bacteria. Consecutive change in confirmation of thrombocytes, or indeed even agglutination, might have been triggered or prevented by certain antibiotic/bug combinations. In conclusion, we have demonstrated that thrombocytes might influence antimicrobial activity, but the effect might vary between different thrombocyte concentrates. Thus, the impact of thrombocytes on antibiotic activity might be easily misinterpreted when only a single experiment is performed. Bacterial growth was not impacted by thrombocytes; in contrast, stability of thrombocytes was highly dependent on the strains used. Thereby, our manuscript provided some preliminary insight in the complex interplay between thrombocytes, bacteria, and antibiotics.

On the bed side, many more factors influence a patient’s outcome and clinical utility of this data is not yet demonstrated. Future studies with prior selected thrombocyte concentrates should be taken in to consideration. Additional, erythrocyte-thrombocyte mixtures could be tested to analyze whether synergistic or antagonistic effects might occur.
